# Vector-Borne Blood Parasites of the Great-Tailed Grackle (*Quiscalus mexicanus)* in East-Central Texas, USA

**DOI:** 10.3390/microorganisms9030504

**Published:** 2021-02-27

**Authors:** Andrew J. Golnar, Matthew C. I. Medeiros, Katlyn Rosenbaum, Justin Bejcek, Sarah A. Hamer, Gabriel L. Hamer

**Affiliations:** 1 Department of Entomology, Texas A&M University, College Station, TX 77843, USA; 2 Pacific Biosciences Research Center, University of Hawai’i at Mānoa, Honolulu, HI 96822, USA; mcmedeir@hawaii.edu; 3 Department of Veterinary Integrative Biosciences, College of Veterinary Medicine and Biomedical Sciences, Texas A&M University, College Station, TX 77843, USA; karosenbaum@gmail.com (K.R.); jbejcek13@tamu.edu (J.B.); SHamer@cvm.tamu.edu (S.A.H.); 4 Schubot Center for Avian Health, College of Veterinary Medicine and Biomedical Sciences, Texas A&M University, College Station, TX 77843, USA

**Keywords:** great-tailed grackle, haemoparasites, invasive species, filarioid nematode, trypanosome, haemosporida

## Abstract

Great-tailed grackles (*Quiscalus mexicanus*) have dramatically expanded into North America over the past century. However, little is known about the blood that parasites they support. Here, for the first time, we document an assemblage of trypanosome, haemosporida, and filarial nematodes co-circulating in invasive great-tailed grackles. Between February and July, 2015, 61 individuals were captured in an urban environment of College Station, Texas. Field microscopy and molecular diagnostics indicate that 52% (24/46) were visually infected with filarioid nematodes, 24% (11/46) with avian trypanosomes, and 73% (*n* = 44/60) with haemosporida parasites, such as *Haemoproteus* (*Parahaemoproteus)* and *Plasmodium cathemerium.* Overall, 87% of great-tailed grackles were infected with blood parasites. Although 50% of individuals hosted parasites from multiple phylum, no patterns of parasite assembly were observed. Results indicate that great-tailed grackles can support a relatively high level of blood parasitism. However, the consequences for avian health remain to be determined.

## 1. Introduction

Populations of great-tailed grackles (GTGR; *Quiscalus mexicanus*) have dramatically expanded into North America over the past century [1,2]. Prior to 1865, GTGR populations were only documented in Central America, Mexico, and the southernmost tip of Texas. Between 1880 and 2000, GTGR increased their breeding range in the United States from an estimated 64,000 km^2^ to more than 3,561,000 km^2^, an annual expansion rate of 3.4% [2]. This contemporary expansion of GTGR populations is likely supported by their unique ability to exploit food resources and safe habitats provided by human-modified environments [2]. For example, adaptive behaviors observed in GTGR include eating dead insects off license plates [3], shadowing farm machinery to collect uncovered invertebrates [4], or roosting in well-lit urban parking lots [2]. The impact of expanding avian species, such as GTGR populations, on avian parasite communities remains to be determined. 

Haemosporida, filarioid nematodes, and trypanosomes are vector-borne blood parasites that persist in a wide range of avian hosts, including some North American *Quiscalus* species [5,6,7,8]. Because of their widespread presence, genetic diversity, and relative ease of sampling, haemosporida and other blood parasites are frequently used to study ecological, evolutionary, and behavioral processes in wildlife systems [9,10]. Although these parasites are geographically and taxonomically widespread, our understanding of avian health, parasite host breadth, and genetic diversity is dependent on field investigations that incrementally advance our understanding of host–parasite dynamics [11,12]. Here we document an assemblage of vector-borne blood parasites that circulate in a population of GTGR, providing baseline data to facilitate additional research into this invasive species.

## 2. Materials and Methods

### 2.1. Study Subjects

Great-tailed Grackles (*Quiscalus mexicanus*) are an invasive bird species that are highly associated with urban and open landscapes, such as agricultural areas, city parks, golf courses, and marshes [2]. Males have a black iridescent plumage with a purplish sheen and a distinctive keel-shaped tail, while females have predominantly dark brown backs, lighter brown underparts, limited gloss on the plumage, and much shorter tails [2]. Populations in the southern US are infamous for roosting in communal groups containing hundreds or thousands of individuals. However, the size and location of roosts vary throughout the year [2,13]. These birds are known carriers of pathogens such as West Nile virus, St. Louis Encephalitis virus, and *Salmonella* [13]. 

### 2.2. Study Area

The study site in College Station, Texas consisted of urban parking lots around several businesses (grocery store, gas station, bank, and restaurants). Inside and around the parking lot contained, there were 55 trees which were utilized as roosting locations by an estimated 2,200 GTGR [13]. Roost locations were primarily southern live oaks (*Quercus virginiana*) that were approximately 8 m in height and located near artificial light [13]. 

### 2.3. Bird Collection

GTGRs were collected from five communal roost locations in urban parking lots of College Station, Texas, USA using modified mist nets as previously described [13]. Sampling events occurred on seven different nights between February and July, 2015. The nets were erected before dusk as the communal birds were staging, approximately 1–4 h before sunset, and run 3–4 h each night. Birds entered the net during staging and roosting activity and birds were extracted shortly after entering the nets. Individual birds were held in a double layer of new paper bags until blood samples were collected. Sexually dimorphic plumage characteristics were used for sex determination and age status (adult or juvenile). Blood extracted by jugular or brachial venipuncture was processed for field microscopy (see below) and molecular diagnostics. The birds were banded with uniquely numbered leg bands issued by the Bird Banding Laboratory of the US Geological Survey and released at the site of capture. Bird trapping and processing was authorized by the Texas A&M University Institutional Committee on Animal Use and Care (2015–0088); a scientific collection permit issued by the Texas Parks and Wildlife Department; and a master bird banding permit issued by the U.S. Geological Survey.

### 2.4. Trypanosome and Filarioid Nematode Infection Prevalence

To determine the infection prevalence of trypanosome and filarioid nematode parasites, approximately 65 μL of whole blood was transferred to a heparinized capillary tube, centrifuged, and screened for the presence of trypanosomes and filarioid nematodes in the field using a 40× compound microscope focused on the buffy coat layer as previously described [14]. After parasite detection, the buffy coat portion from the capillary tube for positive samples was transferred to a microcentrifuge tube for genetic barcoding of parasites. Whole blood not used during field microscopy was separated into serum and clot fractions by centrifugation (14,000× rpm for 6 min) and stored in a −20 °C freezer for molecular diagnostics. 

### 2.5. Haemosporida Infection Prevalence and Lineage Determination

Nucleic acid from avian blood clots was extracted using the Biotek E.Z.N.A tissue DNA kit (Omega Bio-tek, Inc., Norcross, Georgia) after overnight incubation in proteinase K. Initially, DNA from avian blood clots were screened by PCR targeting a 154bp fragment of the haemosporida 16S rRNA gene. Suspect positive samples determined by amplicon size were subjected to a nested PCR that targets a ~590 bp fragment of the mitochondrial cytochrome b gene (*Cytb*) in *Haemoproteus* and *Plasmodium* species [15,16]. Samples that produced PCR amplicons of the correct size for both PCR reactions were considered positive, otherwise they were considered negative.

*Plasmodium* and *Haemoproteus* parasite lineages were determined using genetic barcoding comparing genetic sequences produced from the *Cytb* PCR amplicon to genetic data hosted by NCBI’s Nucleotide Database and MalAvi [17,18]. Briefly, *Cytb* amplicons were purified with ExoSap-It (Affymetrix USB, Cleveland, Ohio) and sequenced using the forward primer of the nested *Cytb* PCR (413F) (Eton Biosciences Inc., San Diego, CA). Sequence chromatographs were inspected individually using 4Peaks version 1.8 (Nucleobytes, The Netherlands) to assess quality by ensuring proper base calls, identifying sequence discrepancies and double peaks. Lineage sequences were aligned in MAFFT [19], trimmed to an even length, and sequence divergence was measured based on a distance matrix generated in Geneious version 9.1.4. Sequences that did not match known *Cytb* lineages with > 99.4% accuracy were considered unique avian malaria lineages [20].

### 2.6. Trypanosome and Filarioid Nematode Phylogenetic Relatedness

DNA was extracted from the buffy coat of a subset of trypanosome (*n* = 4) and filarioid nematode (*n* = 6) samples identified as positive by field microscopy using methods described above. Trypanosome DNA was amplified using a nested PCR targeting a 326bp fragment of the trypanosome SSU rRNA [21]. Filarioid nematode sDNA was amplified using a PCR targeting a 688bp fragment of the conserved nematode mitochondrial cytochrome oxidase 1 (COI) [14,21]. After bi-directional sequencing (described above), sequence chromatographs were assessed for quality and when mismatches were present, IUPAC nucleotide ambiguity codes were substituted.

Aligned forward and reverse consensus sequences were phylogenetically compared to sequences available in NCBI’s nucleotide database. *Thelazia lacrymalis* (GenBank: AJ271619) and *Bodo caudatus* (GenBank: AY490218) were selected as outgroup taxa filarioid nematode and trypanosome datasets, respectively [14]. Datasets were aligned using ClustalW alignment in Geneious version 9.1.4 and trimmed to equal length [22]. Phylogenetic trees were constructed with Bayesian (Mr. Bayes version 3.2.6) and Maximum likelihood (RAxML version 7.2.8) methods in Geneious [23]. Appropriate rates of evolution were selected based on the statistical results of Jmodeltest [24,25].

### 2.7. Polyparasitism Assembly Analysis

Methods described by Janovy et al. [26] were used to determine whether haemoparasite co-infection in GTGR was more or less frequent than expected by chance. Parasite presence/absence resulting from the above diagnostics was used to estimate the frequency of polyparasitism status (no infection, haemosporida (H) parasites only, filarial nematodes (F) only, trypanosome (T) parasites only, H:F co-infection, H:T co-infection, F:T coinfection, and H:F:T co-infection). Observed frequencies of parasite overlap were compared to a null model of expected co-infection frequencies based on the apparent prevalence of parasite colonization detected during this study (*n* = 45) [26]. Significant deviations from the null model was evaluated using the chi-squared statistic [27].

## 3. Results

### 3.1. Bird Processing

In total, 61 GTGR were captured, of which, 59 were adults (49 female, 10 male) and two were juvenile (sex undetermined). Blood samples were obtained from 60 GTGR. One GTGR individual was found dead approximately 380 m from the original sampling site 35 months after capture.

### 3.2. Trypanosome and Filarioid Nematode Infection Prevalence

Of 60 GTGR blood samples, 46 samples yielded sufficient blood volume to screen for trypanosome and filarioid nematodes by hematocrit centrifugation and field microscopy. Of these, 52% (*n* = 24; CI: 37–66%) were visually infected with filarioid nematodes and 24% (*n* = 11; CI: 12–36%) were infected with avian trypanosomes (Table 1). The motility of the parasites in the buffy coat under 40X magnification was recorded for one GTGR (Video S1).

### 3.3. Haemosporida Infection Prevalence and Lineage Determination

The initial PCR targeting the 16S rRNA gene amplified DNA in 47 of the 60 avian blood clot samples. Follo- up nested PCR of the 47 samples produced 44 visible PCR amplicons of correct size. As such, 73% (*n* = 44; CI: 62–85%) of GTGR individuals were determined to be infected with *Plasmodium* or *Haemoproteus* parasites (Table 1). Three reactions failed, which were subsequently determined to be uninfected. High-quality sequences allowed for lineage determination in 38 of these 44 samples. Parasite lineages include *Haemoproteus* (*Parahaemoproteus*) lineage CHI18PA (*n* = 31), CHI22PA (*n* = 6), and CHI18PA/CHI22PA mixed infection (*n* = 1). The *cytB* gene of the *Plasmodium* species detected during this study (*n* = 1) most closely matched *Plasmodium cathemerium* in GenBank with 99% identity (GenBank Accession AY377128.1).

### 3.4. Trypanosome and Filarioid Nematode Phylogenetic Relatedness

**Figure 1:**
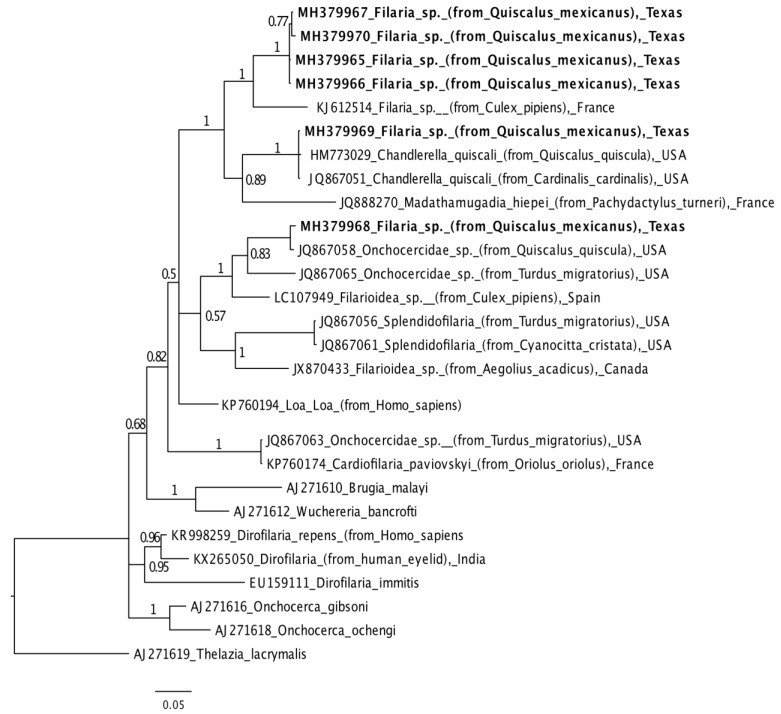
A phylogenetic tree constructed from a 527 base pair segment of filarial nematode DNA inferred using mitochondrial cytochrome oxidase 1 gene sequences from 27 organisms using *Thelazia lacrymalis* (GenBank: AJ271619.1) as an outgroup. Filarial nematode sequences from great-tailed grackles captured in College Station, Texas are listed in bold font.

The GTGR filariod nematode sequence 150622-B04 forms a distinct clade with two *Chandlerella quiscali* sequences isolated from a northern cardinal and a common grackle (*Quiscalus quiscula*) in the USA (Figure 1; GenBank: MH379969). The GTGR isolate 150413-B16 matches an *Onchocercidae* species isolated from a common grackle in the USA with 99% identity and an *Onchocercidae* species isolated from an American robin (*Turdus migratorius*) with 91% identity (Figure 1; GenBank: MH379968). GTGR COI gene sequences 150413-B15 (GenBank: MH379967), 150622-B13 (GenBank: MH379970), 150325-B05 (GenBank: MH379965), and 150331-B15 (GenBank: MH379966) form a monophyletic clade in comparison to other filarioid nematode COI gene sequences (Figure 1). All filarioid nematode COI gene sequences are available on GenBank, with unique sequence identifiers listed in Figure 1 [MH379964-MH379970].

**Figure 2:**
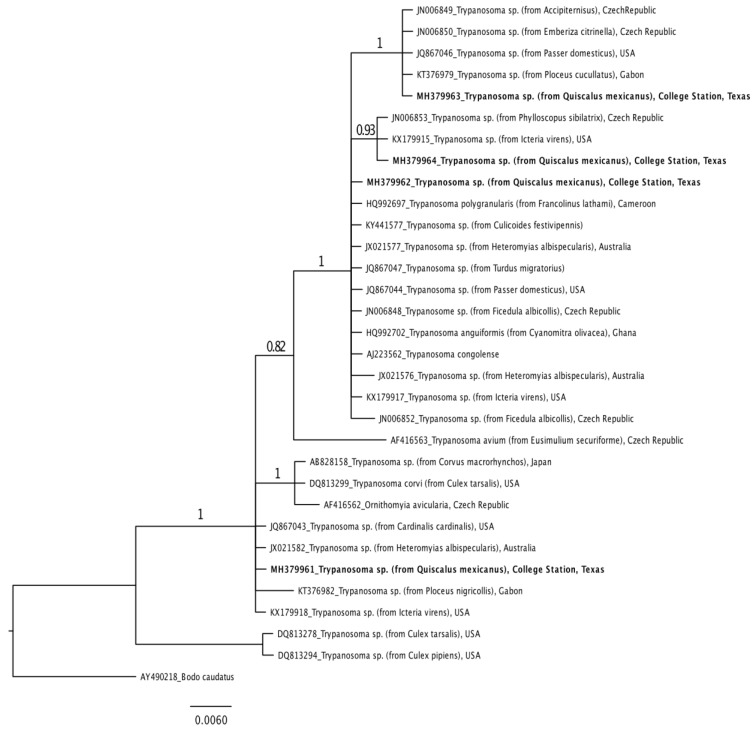
A phylogenetic tree inferred from 219 base pairs of trypanosome 18S RNA gene sequences for 32 taxa with *Bodo caudatus* (GenBank: AY490218.1) as an outgroup. Trypanosome sequences from great-tailed grackles captured in College Station, Texas are listed in bold font.

The SSU rDNA sequence from GTGR 150218-B13 (GenBank: MH379963) is identical to avian trypanosome sequences isolated from a Eurasian sparrowhawk (*Accipiter nisus*) in the Czech Republic, a yellowhammer (*Emberiza citronella*) from the Czech Republic, a house sparrow from the USA, and a village weaver (*Ploceus cucullatus*) from Gabon (Figure 2). These six sequences form a unique clade with posterior probability support of 1. The trypanosome species isolated from GTGR 150622-B07 (GenBank: MH379964) is identical to an avian trypanosome sequence generated from a yellow-breasted chat (*Icteria virens*) captured in the USA and a wood warbler (*Phylloscopus sibilatrix*) captured in the Czech Republic. These three isolates form a clade with 0.93 posterior probability support. The SSU rDNA sequence of GTGR 150325-B12 (GenBank: MH379962) is identical to six trypanosome sequences isolated from a Latham francolin (*Francolinus lathami*) captured in Cameroon, a biting midge (*Culicoides festivipennis*) (unknown location), an Ashy robin (*Hateromyias albispecularis*) from Australia, an American robin from the USA, a house sparrow from the USA, and a collared flycatcher (*Ficedula albicollis*) from the Czech Republic. GTGR 150413-B16 (GenBank: MH379961) is identical to trypanosome genetic sequences isolated from the blood of a northern cardinal (*Cardinalis cardinalis*) in the USA and an ashy robin from Australia. All SSU rDNA sequences of trypanosome species are available on GenBank with unique sequence identifiers listed in Figure 2 [MH379961-MH379964].

### 3.5. Polyparasitism Assembly Analysis

Complete data on haemosporida, trypanosome, and filariod nematode infection status were obtained for 45 individuals. In comparison to a null model, rates of parasite co-infection did not vary from what was expected, suggesting that parasite colonization is not dictated by interactions among different parasite species (Table 2) (X^2^ = 3.9309, *df* = 7, *p*-value = 0.7877).

To determine whether parasite colonization in GTGR was more or less frequent than expected by chance, the frequency of co-infection between multiple parasites was compared to a null model of expected co-infection frequencies based on the apparent prevalence of parasite colonization detected during this study [26]. Significant deviations from the null model were evaluated with the chi-squared statistic. Only blood samples from GTGR that were screened for trypanosome, filarioid nematode, and haemosporida infections were utilized for this analysis (*n* = 45). The null hypothesis (there are no interactions between parasite species dictating host colonization) is rejected if the chi-squared *p*-value is less than 0.05. The chi-squared value for given probabilities = 3.93, *df* = 7, *p* = 0.79.

## 4. Discussion

A comprehensive survey of blood parasites in 388 North American bird species documented that 19.5% of avian species were infected with *Heamoproteus* species, 17.7% with *Leucocytozoan* species, 3.9% with *Trypanosoma* species, 3.8% with *Plasmodium* species, and 3.1% with filarioid nematodes [5]. Here, we document an assemblage of trypanosome, haemosporida, and filarial nematodes in invasive GTGRs. Although no patterns of parasite assembly were observed (Table 2), the detection of blood parasites in 87% of GTGR individuals and co-infection in 50% of birds suggests that GTGR are commonly infected with vector-borne blood parasites (Table 1).

Results suggest that multiple filarioid nematode, *Trypanosoma* lineages, and *Haemoproteus* lineages may be circulating in GTGR populations (Table 1; Figure 1 and Figure 2). Further, results significantly contrast with those collected in Tempe, Arizona, which reported no observations of haemosporida, *Trypanosoma*, or filarioid nematode parasites in 23 GTGR individuals [28]. High levels of parasitism (Texas) and low levels of parasitism (Arizona) may be due to evolutionary (*Q. m. prosopidocola* are predominantly in Texas, while *Q. m nelson* and *Q. m. monsoni* are in Arizona), environmental, ecological, or methodological factors [29]. For example, Avian malaria parasites are known to demonstrate heterogeneity in vector and host compatibility [30,31], thus biting vectors in Arizona may be absent or refractory to these parasites. However, considering vector–host–pathogen interactions are nuanced by physiological (i.e., susceptibility), ecological (i.e., demography, community assembly, space use, interspecific interactions), or environmental (i.e., temperature, resource availability) variation, directed studies are needed to identify what processes drive these differences.

The health consequence of high rates of parasitism and severe densities of microfilariae infection (Video S1) remain unknown. The parasites recovered in this study are generally believed to be non-pathogenic in avian hosts. However, they can elicit severe disease depending on the host–parasite combination or impact long-term demographic dynamics, such as reproductive capacity [9,10,11,32]. Prior studies indicate that *Haemoproteus* lineages CHI18PA and CHI22PA may be generalist parasites of the superfamily Passeroidea and ictarids, respectively [33,34]. Recovery of a banded GTGR individual 35 months after sampling suggests that *Haemoproteus* CHI18PA may not severely influence GTGR mortality. However, without comparing parasite status with metrics of avian health (such as hematocrit values or plumage qualitied), controlled experimentation or long-term population surveillance, the health consequences of high parasitism documented in this study remain unknown.

Considering filarioid nematodes, *Haemoproteus* species, *Plasmodium* species, and avian trypanosomes are vectored by a composite of lice (order Phthiraptera), hippoboscids, mites, mosquitoes, biting midges and black flies (Simuliidae), the centrality of GTGR to a variety of feeding vectors highlights their predisposition to influence vector-borne transmission networks [11,35]. As a follow up to the observations of the current study, we sampled *Culicoides* from this same urban location in College Station, TX in 2016 and documented 10 species with *C. crepuscularis* positive for *Onchocercidae* sp. and *Haemoproteus* sp. DNA [36]. Whether high blood parasitism in GTGR is a result of elevated exposure to vectors or a consequence of GTGR susceptibility, results suggest that GTGR may have a propensity to impact vector-borne parasite dynamics as amplification hosts or sources of parasite spillback [5,37,38,39]. For example, a case report documented a northern crested caracara (*Caracara cheriway*) collected in the same study region and same year as the current study that died due to encephalomyelitis caused by *Chandlerella quiscali* [40]. These filarial nematodes are normally found in grackles, so either this caracara was exposed by consuming an infected bird or via an infected *Culicoides* [40]. In either case, the invasion of GTGR into new regions might alter the parasite community, exposing new hosts to new pathogens. Expanding GTGR populations may provide a valuable model system for exploring relationships between invasive hosts and parasite community dynamics [41,42,43,44].

## Figures and Tables

**Table 1:** Haematoparasite prevalence in great-tailed grackles (*Quiscalus mexicanus*), College Station, Texas, 2015.

Haematoparasite	Genus	Lineage	Count	Sample Size	Est. Prevalence	95% C.I.
Filarioid nematode	-	-	24	46	0.52	0.37–0.66
Avian Trypanosome	-	-	11	46	0.24	0.12–0.36
Haemosporida	-	-	44	60	0.73	0.62–0.85
	* Haemoproteus	-	38	60	0.63	0.51–0.76
	* Haemoproteus	*CHI18PA*	31	60	0.52	0.37–0.63
	* Haemoproteus	*CHI22PA*	6	60	0.1	0.03–0.18
	* Haemoproteus	*CHI18PA/CHI22PA*	1	60	0.02	0–0.05
	*Plasmodium*	*Unclassified*	1	60	0.02	0–0.05
	Undetermined	*-*	5	60	0.08	*-*

* Haemoproteus (Parahaemoproteus) species; C.I. = confidence interval; “-“ indicates absence of data

**Table 2:** Co-infection of great-tailed grackles (*Quiscalus mexicanus*), College Station, Texas, 2015.

Infection Status	Sample Size	Expected	Observed	Estimated Portion of Population	95% Confidence Interval
No infection	*45*	4	*6*	0.13	0.03–0.22
Haemosporida sp. (H)	45	12	9	0.20	0.08–0.32
Filarial Nematode sp. (F)	45	5	4	0.09	0.01–0.17
Trypanosome sp. (T)	45	1	2	0.04	0–0.10
*H:F* co-infection	*45*	13	*16*	0.36	0.22–0.50
*H:T* co-infection	*45*	4	*4*	0.09	0.01–0.17
*F:T* co-infection	*45*	1	*0*	0.00	0
*H:F:T* co-infection	*45*	4	*4*	0.09	0.01–0.17

## Data Availability

Genetic sequence data are publicly available in the GenBank repository.

## Supplementary Materials

The following are available online at https://www.mdpi.com/2076-2607/9/3/504/s1, Video S1: Motile microfilariae in blood buffy coat of great-tailed grackle (*Quiscalus mexicanus*).
